# SHACLens: a visualization workflow for SHACL violation exploration in knowledge graphs

**DOI:** 10.3389/fbinf.2026.1756507

**Published:** 2026-03-23

**Authors:** Christian A. Steinparz, Andreas Hinterreiter, Labinot Bajraktari, Vitaly Sedlyarov, Markus J. Bauer, Thomas Zichner, Marc Streit

**Affiliations:** 1 Visual Data Science Lab, Johannes Kepler University, Linz, Austria; 2 Boehringer Ingelheim RCV GmbH & Co KG, Vienna, Austria

**Keywords:** data curation, down-projection, knowledge graphs, large language models, LLM interfaces, machine learning, visual analytics, visualization

## Abstract

**Introduction:**

Validating large knowledge graphs with the Shapes Constraint Language (SHACL) often yields violation reports too large to interpret and trace to root causes, especially in industry-scale datasets such as pharmaceutical omics pipelines.

**Methods:**

We present SHACLens, an interactive visualization workflow—developed with a major pharmaceutical partner—that links ontology, instance data, and violation reports across multiple coordinated views. We contribute a practitioner-informed workflow co-designed with pharmaceutical data-analysis experts. A Node-Link View combines ontology and groups of equivalent violations, a projection view reveals clusters of nodes with similar errors, a LineUp table combines instance data with violation information, a Class Tree offers a class-hierarchy overview, and an integrated LLM assistant provides contextual explanations and can operate the system via natural-language commands.

**Results:**

Within this workflow, selections and filters propagate across views, exposing co-occurring errors and their likely upstream causes. Analysts iteratively identify violation clusters, inspect correlations, and trace the detailed cause of errors.

**Evaluation and implications:**

We evaluated SHACLens through an iterative expert-in-the-loop design process with the partner team and a qualitative study on a transcriptomics dataset containing 5,203 violating nodes with the same experts. In this study, SHACLens efficiently surfaced repeated sets of errors due to missing objects and schema inconsistencies, supporting goal-oriented analysis and serendipitous findings.

## 1 Introduction

Knowledge graphs (KGs) have emerged as indispensable platforms for capturing and structuring knowledge from complex domains. They enable users to define domain schemata in a standardized language and are grounded in well-established logical formalisms that support reasoning and inference. Recently, KGs have become widely recognized and are used by large tech companies (Noy et al., 2019) and in various academic and community projects (e.g., Wikidata (Vrandečić and Krötzsch, 2014)). An additional advantage of capturing data in KGs is the ability to verify the correctness, completeness, and consistency of the data. This is possible by checking whether the data adheres to specific constraints, such as those defined by the Shapes Constraint Language (SHACL). This verification yields a detailed violation report, which includes information about the violating nodes and broken constraints.

However, the scale of KGs is often immense and in the most extreme cases can reach up to tens of billions of entries (Noy et al., 2019). Additionally, the relationships between violations, individual data instances, and data classes—which are defined in a so-called *ontology*—can be complex. Thus, manual analysis of the violation data and the KG quickly becomes arduous or is no longer feasible. In a recent paper on the challenges and opportunities for visualization in the context of KGs, Li et al. (2024) mention data quality issues among the central challenges of working with KGs. In this context, Lit et al. see a need for innovative approaches that go beyond merely using query languages. This claim resonates not only with previous literature (Yahya et al., 2016; Koutrika et al., 2014), but also with the experience and insights of our domain expert collaborators.

We collaborated with domain experts from the pharmaceutical industry, who specialize in generating, processing, and analyzing large-scale biological datasets for applications in the area of drug discovery. These datasets include various types of biological information, such as proteomics (the study of proteins) and transcriptomics (the study of ribonucleic acid or RNA transcripts), which are collectively referred to as omics data. Our collaborators use KGs to capture the metadata of their ingested omics studies, and use SHACL (Knublauch and Kontokostas, 2017) to validate the structure and integrity of the data. Due to the nature and size of their datasets, our collaborators have struggled to efficiently analyze constraint violation data based only on textual reports and queries.

**FIGURE 1:**
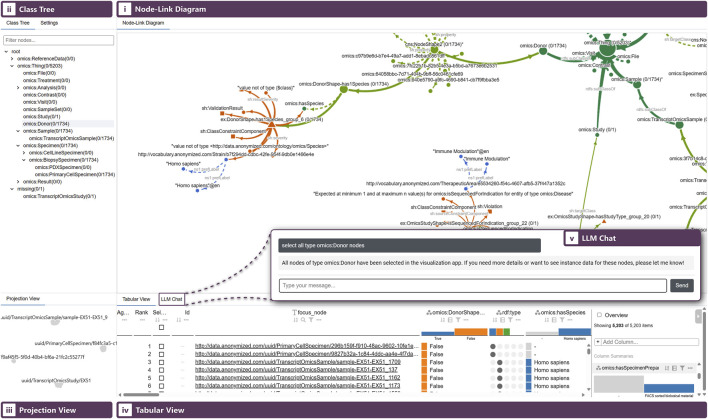
The SHACLens dashboard consists of five synchronized views: (*i*) Node-Link Diagram (NLD) connecting ontology, SHACL constraints, and grouped violation reports. (*ii*) Class Tree showing the ontology’s rdfs:subClassOfhierarchy. (*iii*) Projection View showing focus nodes that cluster due to similar or equivalent violated constraints. (*iv*) Tabular View (LineUp) showing a tabularized combination of violation report and instance data. (*v*) LLM Chat used to control the dashboard selections and get explanations about the data.

In this work, we address the challenge of analyzing the relationship between violations and KGs when large SHACL violation reports are infeasible to interpret manually, and we facilitate the linking of violations to instance data to identify upstream causes for actionable error correction. To this end, we propose SHACLens (see Figure 1), an interactive dashboard that integrates visualization techniques with machine learning approaches. Specifically, SHACLens supports users in: (*i*) understanding overall distributions and co-occurrence of violations; (*ii*) tracing the graph-topological paths that link members of the ontology to SHACL violations; (*iii*) reducing scalability issues by grouping thousands of equivalent violations; and (*iv*) relating violations to instance-level properties and their distributions to identify the patterns that trigger errors. SHACLens allows users to navigate from classes to constraints and from grouped violations to the implicated instance data in multiple linked views. Users can interact with all parts of our solution traditionally, that is, through mouse actions and text-based filtering, or using the integrated large language model (LLM) chat interface. The chat supports users, for example, by answering questions about the data, explaining the displayed content, or automatically selecting and visually highlighting relevant information. We showcase the benefits of SHACLens in a case study with domain experts, and describe how it helped them to efficiently uncover inconsistencies in their data from a previously overwhelming volume of SHACL test results.

## 2 A knowledge graph primer

As mentioned, KGs have become widely used in industry, academia, and community projects as a means of capturing data based on flexible schemata, while providing the ability to check the data for completeness and integrity. While the term “knowledge graph” has been used in a variety of ways (Ehrlinger and Wöß, 2016), in this paper, we use the term KGs for data structures that adhere to the concepts defined in the W3C RDF 1.1 standard (World Wide Web Consortium, 2014). This standard provides a framework for describing data and ensuring its interoperability.

For the remainder of this work, we view KGs as being composed of two parts: the *ontology* and the *instance data*. An *ontology*, in some contexts also referred to as “schema”, is the formal representation of the knowledge within a specific domain. It captures the logical theory of the domain in the form of classes, properties, and their relationships. An ontology employs an axiomatic language to define these concepts, and it can have a range of expressivities depending on the constructors used. In this work, we focus on lightweight ontologies defined using the Resource Description Framework Schema (RDFS) (Brickley and Guha, 2014). *Instance data* on the other hand, captures factual information about specific entities and their relationships within the domain of interest. The recorded entities and their relationships are represented as instances of classes and properties, as defined in the ontology that models the domain. One standard for describing instance data is the Resource Description Framework (RDF), which captures facts in the form of (*subject*, *predicate*, *object*) triples. Viewed as a graph, the subject and object represent nodes, while the predicate corresponds to the edge that connects them.

For example, the fact that Boromir’s ancestry is the Men of Gondor can be expressed in a knowledge graph through the triple (*Boromir*, *hasAncestry*, *Men of Gondor*). Furthermore, we might want to capture that Boromir is a character in Tolkien’s legendarium. This can be achieved with the triples (*Boromir*, *is a*, *Character*). These two triples are examples of instance data. In an associated ontology, “Character” is defined as a subclass of “Thing”, which is the most general class. Further relationships could be added, such as a class for secondary characters, which could be defined as a subclass of “Character”. The blue and teal parts to the left and bottom left of Figure 1 show a very small example ontology and some minimal instance data. In practice, entities in KGs are encoded using long Uniform Resource Identifiers (URIs) to ensure their uniqueness. To manage the lengthy identifiers, the concept of a *namespace* is employed to capture common prefixes shared across related terms. For example, the class we previously introduced as “Character”, in practice, would be referred to as lotr:Character in a KG. Here, the prefix lotr: is the namespace substring that refers to all terms related to the Lord of the Rings. This convention also helps users to better identify those terms uniquely within a broader context.

To ensure the quality and consistency of data within KGs and their alignment with the established ontologies, the Shapes Constraint Language (SHACL) is employed. In SHACL, the term “shape” refers to a constraint or condition that RDF data must satisfy. SHACL allows users to validate the structure of RDF graphs, ensuring that they adhere to specific patterns and rules. For example, one constraint for the data in our knowledge graph might be that each character must have an ancestry. This could be realized through a SHACL *shape* that ensures that each entity classified as “Character” has exactly one “hasAncestry” relationship with an entity of class “Ancestry”. In the minimal example illustrated in Figure 1, this is realized by a *node shape* defined for the class “Character” and a *property shape* associated with it.

Instance data modeled and based on a specific ontology can then be automatically tested against these SHACL constraints to identify any deviations or errors. This process generates another KG about the occurrence of violations in the data, typically called *violation report*. Domain experts use this violation graph to detect and resolve data inconsistencies. The violation knowledge graph captures in detail which violations were triggered by connecting violating nodes—typically referred to as *focus* nodes—with the violated shapes. In our example in Figure 1, a violation is triggered for Frodo Baggins, because no ancestry has been defined for Frodo. Boromir, whose ancestry is properly defined as “Men of Gondor”, fulfils the property shape and does not trigger a violation. Fixing the reported violation requires the introduction of Frodo’s ancestry, “Hobbits”, which needs to be defined as an “Ancestry” and connected to Frodo by a “hasAncestry” link.

Even for rather small knowledge graphs, parsing the violation report and understanding why violations are triggered can be a challenging task for humans. The combined complexity of the ontology, instance data, SHACL constraints, and violation data, therefore, presents an interesting visualization challenge.

## 3 Related work

Verifying KGs using SHACL constraints falls into the domain of *data curation*. Data curation involves assembling, organizing, and maintaining datasets to ensure they remain accessible and usable. The importance of data curation is reinforced within the KG community through the widespread adoption of the FAIR Guiding Principles (Wilkinson et al., 2016). The process of ensuring that KGs match specific constraints is an example of *verification* (Huaman and Fensel, 2022). Especially for industry-scale KGs, manual verification becomes infeasible Noy et al. (2019). As a result, a multitude of frameworks and tools have been developed to facilitate data curation (Simsek et al., 2021; McCusker et al., 2018; Malik et al., 2020; Hoyt et al., 2019), including some works that concern themselves with SHACL (Spahiu et al., 2018; Simsek et al., 2021; Figuera et al., 2021). However, such tools typically do not focus on supporting verification through visualization.

In our work, we employ KG visualization to support the analysis of SHACL violation reports. KG visualization is a subfield of *graph or network visualization*. Depending on how triples are interpreted, KG visualization can be viewed more specifically as an example of *multivariate network visualization* (see Nobre et al. (2019) for a survey of techniques). In the semantic web community, KG visualization is often discussed in the context of *linked data visualization* (Po et al., 2020; Klímek et al., 2019; Bikakis and Sellis, 2016). In the following, we first discuss previously published insights and challenges related to the intersection of KGs and visualization, before discussing works that specifically address SHACL and KG verification.

### 3.1 Knowledge graphs & visualization workflows

Li et al. (2024) studied the challenges and opportunities of using visualizations alongside KGs in practice. We consider this study to be exceptionally relevant and useful when designing a KG visualization workflow, and several of the presented findings resonate with our experience from collaborating with domain experts. One of the insights provided by Li et al. (2024) is that the use of query languages alone is insufficient for effective and intuitive KG analysis workflows. This view aligns with additional literature in this area (Yahya et al., 2016; Koutrika et al., 2014) and is shared by our domain-expert collaborators.

According to Li et al. (2024) and Dudáš et al. (2014), node-link diagrams (NLDs) are critical in the context of KGs for certain user tasks related to analyzing topological structures, as long as rich interactions are provided. They list specific interactions such as filtering, condensing, or collapsing substructures, and cross-linking with other views, which are all supported by SHACLens. The complexities of specific use cases, such as analyzing large quantities of violation report data, necessitate customized visualizations (Li et al., 2024). In this context, Dudáš et al. (2014) provide a categorization of visualization tools for specific KG-related use cases. We discuss some specific related works in this context in the following section.

Finally, one key insight reported by Li et al. (2024), which is central to the design of our solution, is the importance of enabling users to directly “ask the KG questions” to facilitate data discovery and exploration. This insight highlights a shift towards interfaces with intuitive user interactions, eliminating the need for learning complex query languages. Our solution addresses the idea of allowing users to ask questions in a quite literal form: users can directly interact with the data and visualizations through a GPT (Radford and Narasimhan, 2018; OpenAI et al., 2024) API via natural language.

Katifori et al. (2007) present a survey on ontology visualization methods, stating that different visualizations are appropriate for various specific user needs; Dudáš et al. (2018) likewise survey ontology visualization approaches. While some more general ontology visualization methods could in theory be applied to constraint-based analysis tasks, most ontology visualization tools and workflows do not consider SHACL explicitly. This includes many works discussed in the surveys as well as a multitude of other academic and commercial solutions (Storey et al., 2001; Falconer, 2010; Víctor Méndez, 2024; Sintek, 2007; Horridge, 2010; Kunowski and Boiński, 2012; Alani, 2003; Bosca et al., 2005).

### 3.2 SHACL and curation-focused KG visualization

Only few works in the visualization literature tackle verification and constraint-checking of KGs, and even fewer have a specific focus on SHACL violation reports. OWLGrED (Liepinš et al., 2014) partially supports the analysis of constraints and violations, but does not go beyond minimally annotated UML-style diagrams. The VizCurator tool by Ghadiri Bashardoost et al. (2015) provides a suite of linked views for curating RDF and similar open data. It was developed for understanding large schemas, temporal semantics, and refining schemas, but does not directly interface with SHACL violation reports. The commercial tool TopBraid Composer (TopQuadrant, Inc, 2019) is described as an ontology modeling and application development environment. Its constraint checking is provided through SPARQL Inferencing Notation (SPIN) rules and a SHACL verification view that lists failures. Parts of the SHACL feature set of TopBraid Composer were still experimental at the time of publishing, and we found no evidence of capabilities needed for large-scale violation analysis.

The Master’s thesis by Alom (2022) introduces SHACLViewer, a tool that lets users load individual SHACL definitions from JSON files and integrate them into a graph format. SHACLViewer provides a visualization of constraints within an ontology utilizing an NLD as well as a tree view. However, it does not provide a visualization for instance data or linked violation reports. SHAPEness (Paciello et al., 2023) is closely related to our work. It is a multi-view tool designed for editing, structuring, and verifying RDF graphs against SHACL constraints. While SHAPEness alerts users of problems in individual property fields, its interface does not appear to be scalable, and it does not focus on the large-scale analysis of violation patterns.

The SHACL Dashboard by Mäkelburg et al. (2025) is a recent attempt at facilitating the SHACL-based KG verification workflow with an interactive visual interface. The SHACL Dashboard provides a web-based interface focusing on aggregated report statistics that summarize violations across various dimensions. While the authors frame the dashboard as a support tool for corrective action, the focus of the supported analysis is mostly on the evaluation of runtime behavior and query scalability. Its primary outputs remain report-centric counts and individual violation listings, which—while useful for overview purposes—do not directly facilitate the analysis of more complex violation patterns and their relation to the underlying data.

In summary, there remains a significant gap in the literature concerning the development of visualization tools that not only display SHACL constraints within the ontology, but also visualize violation reports in combination with the violating instance data in a scalable manner. SHACLens is our attempt to fill this gap by following a visual analytics approach, linking ontology, shapes, grouped violations, and instance data through diverse, coordinated visual representations. Moreover, to the best of our knowledge, no existing solution addresses this task while leveraging state-of-the-art technologies such as machine learning and large language models to enable intuitive user interaction.

## 4 Problem characterization

As outlined above, to the best of our knowledge, there are no visualization tools optimized for analyzing SHACL violation data that can address our collaborators’ use case. In this section, we first describe our target users and the general workflow they employ in the absence of a visual solution. We then discuss why such a visual solution would be beneficial to them. Finally, we extract a number of user tasks and introduce additional design requirements for our solution.

### 4.1 Users

The target users of our visualization solution for analyzing SHACL violation data are a heterogeneous group of data workers at a large pharmaceutical company working with extensive multi-omics knowledge graphs. These users can be broadly categorized by their roles and areas of expertise into data *providers* and data *quality analysts*. Our solution aims to address violation analysis tasks that are shared by both types of users.

Data *providers* are responsible for entering new instance data into the KG. In the case of our collaborators, these are the metadata of omics studies. While the data providers have a high level of domain expertise about these studies, they have varying degrees of familiarity with the KG technology stack. The KG persona classification by Li et al. (2024) does not explicitly cover this type of user. While a subset of data providers makes use of KG-based insights, and can therefore be considered KG “consumers”, most are not involved in the KG creation in sufficient detail to be considered true KG “builders”. According to the classification of data science roles by Crisan et al. (2021), the user group of data providers can be categorized somewhere between “research scientist”, “technical analyst”, and “moonlighter”.

Data *quality analysts* are responsible for resolving errors in the KG and for making sure that potential updates to the ontology are smoothly integrated. This user group possesses detailed knowledge of the various KG technologies used, but may lack some domain knowledge. This aligns well with the “analyst” persona as defined by Li et al. (2024). In terms of data science roles, data quality analysts can be described as “data shapers” or “data engineers” (Crisan et al., 2021).

### 4.2 Source of errors

Most errors that lead to violations are introduced during input time. Data providers typically upload tabular data from spreadsheets or CSV files via a data entry tool. In theory, errors should be captured and resolved before or during this stage. In practice, this is not feasible for two reasons: One, data input is decentralized across sites using different tools that may not be able to expose the most up-to-date ontology and constraints to users. This makes it difficult, for example, to disallow entries that violate constraints directly in a web form. Two, data providers have varying degrees of understanding of violation data. They might not be able to pinpoint the sources of errors when presented only with the violation report.

Changes to the ontology are another potential source of error. Data quality analysts typically perform these updates in an attempt to refine the existing ontology, consolidate different data sources, or prepare the KG for the inclusion of new data modalities.

### 4.3 Curation workflow

To better understand the user tasks and requirements for our visualization solution, we analyzed the existing workflow of our collaborators. To this end, we conducted two multi-hour workshops with two data quality analysts and two scientists in leading positions, in which they explained and showcased their current workflow and discussed pain points. In the following, we break down their workflow into multiple *actions*:

- *Pick a violation*—To attempt to fix an error, data providers and quality analysts first pick a violation from the violation report. They access the violation data in the form of an HTML report in a web app, which exposes the error message as defined in SHACL and the number of triggered violations. Data providers pick violations based on the importance of information that might be missing, with less focus on structural violations. Data quality analysts, in addition, may use counts and other statistics to pick violations.
- *Locate the error*—Next, users attempt to identify the source of the error, which varies greatly in complexity. Violations can result from simple missing but required data entries, but can also be the result of complex patterns of SHACL shapes with potentially obscure names resulting from automatic constraint processing. We showcase an exemplary complex pattern in our guiding dataset in Section 4.5, where a single faulty entry of an instance of one class leads to a cascade of violations that ultimately affect many instances of other classes. Because data providers typically do not have the KG expertise required to write queries, such underlying effects are almost impossible to understand for them. Data quality analysts possess the necessary KG expertise to iteratively write queries until the resulting number of items matches the number of constraint violations listed in the report. However, this is done in a purely text-based interface and complicated by the difficulty of understanding the patterns and interactions between violations in the absence of any visual aids.
- *Fix the error*—If data providers could correctly locate the source of an error, they can attempt to fix the error through edits in the input tool. Otherwise, the error remains in the backlog of errors for data quality analysts to fix. Data quality analysts can develop queries to fix errors identified by themselves or by the providers, as long as that potentially missing information is available. Finally, data quality analysts may rerun the validation workflow to verify any fixes applied.

### 4.4 Tasks & requirements

Our solution aims to help users identify violations and locate the underlying error sources. We thus address the first two user actions listed above. We derived the following tasks from these actions, which were continuously refined in the early design iterations.

T1 Access ontology–A visualization solution needs to make the ontology accessible. This is especially important for data providers, who typically do not have easy access to class definitions and their relationships.
T2 Locate violation in ontology–It must be easy for users to discern where raised violations fall within the ontology’s structure. Often, violations triggered by a specific focus node are actually caused by a different entry of the KG or an issue with the ontology.
T3 Group nodes by violations–Often, many focus nodes are associated with the same types of violations, for example, if many nodes have a relationship with a single node whose type is incorrectly defined. Grouping such violation patterns helps reveal problematic parts of the KG and enables the correction of many errors simultaneously.
T4 Find co-occurring violations–Violations can co-occur. For example, a node violating a node shape may also be associated with a property shape violation. Allowing users to find such co-occurrence patterns should help them understand the violation reports better and find potential fixes more effectively.
T5 Access property distribution–Users are often interested in the distribution of properties and/or classes among focus nodes that cause violations, as these distributions can help identify the source of the error.
T6 Hide cluttering information–Violation reports often contain many uninteresting entries, such as timestamps or auto-generated keys. Allowing users to flexibly hide specific parts of the ontology and/or violation data is necessary to avoid information overload that is not required to understand or fix the violations.

While these tasks mainly support locating and understanding violations, we also partially support the process of developing correction queries through the LLM chatbot described in Section 5.5. Finally, we also identified two technical requirements for our visualization solution: One, the solution must combine the violation data with the ontology and the relevant instance data (e.g., to address T2 and T5). And two, users should not be expected to know how to write SPARQL queries.

### 4.5 Guiding example dataset

To better illustrate the structure of our collaborator’s data and typical violations, we introduce a minimal guiding example dataset. We chose to create a Lord of the Rings (LotR) KG toy dataset, which enables us to illustrate typical violations without having to explain the complex domain-specific ontologies used by our collaborators. We also use this toy dataset as a guiding example for explaining the design of our solution (see Section 5). Similar to the minimal example shown in Figure 1, this guiding example contains LotR characters (e.g., Frodo, Aragorn) and ancestries (e.g., Hobbit, Men of Gondor). To allows us to illustrate more complex violations, we also introdcue locations (e.g., The Shire, Minas Tirith), regions (e.g., Eriador, Gondor), and the continent Middle-earth. Several SHACL constraints govern relationships in this KG: each character must have a defined ancestry and home location; each location must belong to a valid region; and each region must belong to a valid continent.

However, due to a missing link between Middle-earth and the class lotr:Continent, all regions are rendered invalid. The problem cascades down, causing violations in locations and ultimately characters. This cascade is an example of the co-occurrence of violations described in T4. Some characters lack a definition for their home. Additionally, the definition of the ancestry lotr:Hobbit is missing, and Hobbit characters have no lotr:hasAncestry predicate link to such an object. The pattern of all Hobbits raising ancestry violations is an example of problems described in T3 and T5. We do not claim that a cascading chain of SHACL constraints necessarily adheres to best practices in defining ontologies. However, such problems may arise in real data, and therefore, it is in the interest of the design to enable the identification of such issues.

Below is a list of all relevant violations in our example dataset:

- *Hobbit not defined* (lotr:CharacterShape-hasAncestry)— Each character must have at least one ancestry. Frodo’s ancestry should be lotr:Hobbit. However, lotr:Hobbit is not defined at all.
- *Middle-earth not a continent* (lotr:RegionShape-isInContinent)— Every region must be on a continent. Rohan is in Middle-earth. However, lotr:MiddleEarth is not defined as a lotr:Continent.
- *No valid regions cascade* (lotr:LocationShape-isInRegion)— Every location must be in a region. Edoras is in Rohan. However, Rohan and all other regions are invalid because they all violate lotr:isInContinent.
- *No valid locations cascade* (lotr:CharacterShape-hasHome)— Every character must have at least one home. Frodo’s home is The Shire, but The Shire is invalid, as are all other locations. They all violate lotr:isInRegion because all regions are invalid.
- *Missing home entries* (lotr:CharacterShape-hasHome)— Some characters are missing a home definition altogether.

While we developed this dataset specifically to explain our design, we discovered similar types of violations in our case study of real data provided by our collaborators.

## 5 Design

We designed SHACLens, an interactive dashboard composed of multiple coordinated views to address the user tasks and requirements outlined in Section 4.4. The design of SHACLens was iteratively refined in close collaboration with the domain-expert users. To this end, we conducted four additional multi-hour workshops and multiple follow-up meetings with two data quality analysts. We also organized a 1-h demo and feedback session with a group of data providers about halfway through the iterative design process to ensure that our design did not steer away too far from the needs of the broader user group.

Our solution consists of (*i*) a Node-Link Diagram (NLD) visualizing ontology, constraint, and violation data, (*ii*) a Projection View showing a scatterplot of violating focus nodes based on dimensionality reduction, (*iii*) a Tabular View showing focus node details, (*iv*) a Class Tree View showing the RDF class hierarchy, and (*v*) an integrated LLM Chat interface. Each view interacts seamlessly with the others. Selections made in one view propagate to all other views, which provides multiple complementary perspectives on the data. We discuss each view and the interactions between them, utilizing our guiding example dataset described in Section 4.5. We further explain how each view addresses the user tasks identified in Section 4.4. A prototype of SHACLens is available at https://shaclens.caleydoapp.org/.

### 5.1 Node-link diagram

We employ an NLD in SHACLens to allow users to understand the context of SHACL shape definitions and their placement within the ontology (T1), and to pinpoint SHACL constraint violations (T2). To support these tasks while maintaining a clean layout, equivalent violations are grouped by default (T3). Users can preview the node and edge expansions before committing to structural changes, expanding connections between ontology elements and violations only on demand (T6). The view is built on a transformation of the ontology, SHACL node/property shapes, and aggregated violation report groups which make up the nodes and edges presented.

We adhere to a similar design as the one used for the conceptual Figure 1. As requested by a data quality analyst, namespaces are visually differentiated using glyph shapes: squares represent SHACL terms such as sh:Violation; circles were chosen as the most frequent shape, as they denote the dominant ontology-specific namespace, for example, lotr: or omics:; triangles identify violation exemplars in the ex: namespace; diamonds are used for owl: terms; and hexagons represent literals and remaining namespaces.

**FIGURE 2:**
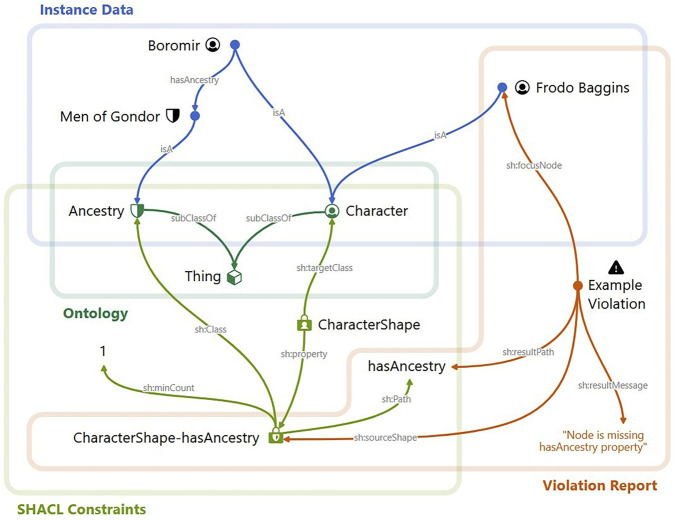
A minimal example that shows the relationships between an ontology, some instance data, SHACL constraints, and a violation report. The report lists only a single violation that was triggered because the data lacks a required “Ancestry” for Frodo Baggins.

Because the data is split across four components from three RDF files, and distinguishing them is necessary to interpret how constraints attach to classes and how they produce violations, we color-code each component. The ontology is encoded in green (Figure 2A). Instance data is encoded in blue and, for scalability, is shown in the NLD only when it appears as a direct object of a violation (Figure 2B). SHACL shapes are encoded in a lighter shade of green to distinguish them from ontology elements despite sharing the same source file (Figure 2C). Violation report data is encoded in red (Figure 2D).

Node radii and edge widths scale with cumulative violation counts, as shown by the number appended to each label. This makes it easy to see where violations are concentrated and how they flow through ontology classes, shapes, and reports. If two incident nodes have different counts, the connecting edge is drawn with a tapered thickness from source to target.

We also encode data in the line style of edges. Solid edges mark the essential path from classes to node shapes to property shapes to grouped violations and their children. All other relations are dashed. Furthermore, edges are rendered as curved splines. While the literature reports mixed and contradictory results on their legibility, often studied in layouts dissimilar to ours, we found curved edges easier to read and follow for the problem and data at hand. We did not formally evaluate this effect, and more conclusive quantitative studies across diverse graph layouts and tasks are needed to clarify these inconsistencies. For selection highlights, we fade non-selected graph elements instead of using the initially considered black outlines around selected elements. This preserves the original encodings for the highlighted subgraph and improves readability.

We apply semantic zoom to node size, line width, and label size so that they remain constant in screen space, while only distances between them change on zoom. To mitigate label clutter, we use cartographic labeling where labels of lower-priority concepts are hidden on collision. We prioritize node labels over edge labels and prefer higher cumulative violation counts over lower ones, which maintains the visibility of violation hubs when zoomed out, while less important labels drop out first. We optimize label readability by giving both node and edge labels a white outline, while rendering edge labels slightly smaller and in gray as they are typically less relevant.

**FIGURE 3:**
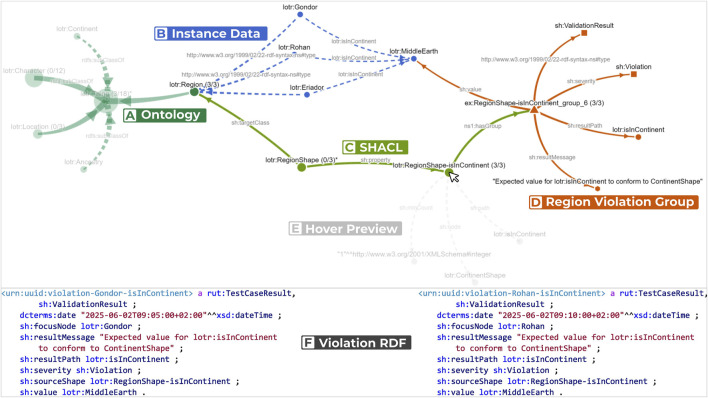
Node-Link Diagram View with an expanded and highlighted subtree for a violation of the “Region must be in continent” constraint. **(A)** Ontology context. Most nodes are faded due to the current selection, except the relevant lotr:Regionclass. **(B)** Instance data. The NLD includes only instances that appear as direct objects in violations. lotr:MiddleEarthis not connected to lotr:Continent, while the regions show their respective connections. **(C)** SHACL shapes. The view shows the node shape and the corresponding property shape. **(D)** Grouped violation details. The group aggregates all three focus nodes that violate RegionShape-isInContinent. **(E)** Hover preview. Mouse-over previews additional children that could be expanded for the isInContinentconstraint. **(F)** Violation RDF. The RDF listing for two of the three regions matching the violations reported in the group in **(D)**.

Violations are grouped when they share the same shape, message, and object value, and rendered as a single exemplar annotated with its group size label. Figure 2D shows such a violation group by the regions Gondor, Rohan, and Eriador. Its count label 3/3 indicates that all violations from this group are currently selected. Figure 2F presents the raw RDF for two region violations to illustrate the equivalence basis for the grouping. Upon hovering on nodes, users are presented with semi-transparent previews of candidates for expansion (Figure 2E). Once a node is expanded, the force-directed layout is incrementally updated. Users can expand/collapse subtrees, drag nodes, pan, and use a context menu to reset the view or selections. The ability to drag nodes makes it easy to rotate the graph as needed. We provide a dedicated action for expanding all violation groups related to a class (and *vice versa*) to surface connected shapes and their groups in a single step. Because collapsing parts of a KG based on parent or child relations can hide relevant context and proved cumbersome in practice, we instead allow users to draw a lasso for selection and hide the selection with a single Delete keystroke, which is fast and precise. Figure 3 demonstrates an overview of the guiding example with all violation paths expanded. Selections in the NLD highlight corresponding classes in the hierarchy, reveal focus nodes in the Projection View, and filter the Tabular View to the selected focus nodes.

As stated above, we designed the NLD to only show grouped violations. While this design choice helps avoid visual clutter, the aggregation hides individual nuances such as focus-node specifics. NLDs tend to become dense and hard to read once more than a few hundred nodes are visible, which also limits the practical scale of our NLD. For this reason, we restrict it to the ontology, shapes, and grouped violations, and we keep only those instance nodes that are direct objects of violations, excluding all other instance data that would otherwise scale to thousands or millions of nodes. We introduce a dedicated Tabular View to allow users to interact with instance details.

**FIGURE 4:**
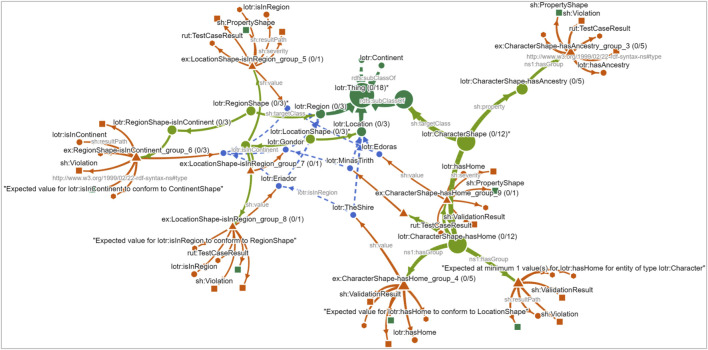
The NLD showing a full expansion of the entire guiding example.

Another difficulty users face is that the directionality between shapes and classes in SHACL can be unintuitive when navigating from class to violation, as indicated in Figure 4. Traditionally, in NLD visualizations, navigation is based on *expand parents* and *expand children* actions. The most relevant navigation in the NLD that users need to perform frequently is the navigation from a class to one of its violations. However, in SHACL, users would need to navigate from a Class to its parent NodeShape, then to the child PropertyShape, and then to the parent Violation. In the design phase, these parent-child directionalities proved to be confusing and tedious for users, resulting in a loss of orientation. To address this, in our NLD, users can use previews and the direct “expand connected shapes/classes” action to mitigate this problem.

### 5.2 Class Tree View

The Class Tree View provides a class-based entry point and an overview of the violation distribution (T1, T2). It shows the class hierarchy with per-class violation counts and supports selection and filtering. Selections both drive and reflect the state of the other views. The view operates on the ontology’s class hierarchy via rdfs:subClassOf and counts of violating focus nodes per class. It also exposes a *Missing* bracket for instances whose declared class does not appear in the ontology. Classes are shown as an indented tree. Node labels reuse the same ClassName (n/m) notation introduced for violation groups in the NLD. Selections are visually highlighted. Users can click a class to select all associated focus nodes, type a substring to jump to a class, and auto-expand only branches relevant to the current selection.

**FIGURE 5:**
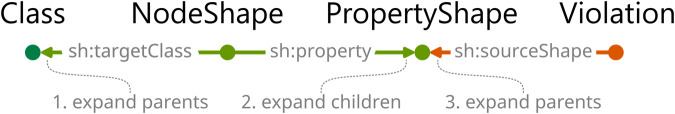
SHACL directionality confuses class-to-violation navigation under traditional NLD expands.

**FIGURE 6:**
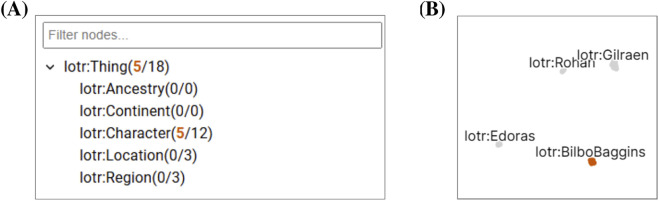
The Class Tree View and Projection View in SHACLens for a synchronized selection. **(A)** The Class Tree View shows the current selection of five Hobbit focus nodes in the context of the classes defined in the ontology. In more complex scenarios, only branches relevant to the selection are expanded automatically. Seven remaining characters, three locations, and three regions also caused violations but are not selected. **(B)** The Projection View shows all projected focus nodes in a scatterplot. The user selected a cluster of Hobbit nodes that all share a violation pattern.

Figure 6A shows the count labels and selection highlighting for a selection of Hobbits. Figure 5ii further demonstrates expansion of relevant branches in a partially collapsed hierarchy. Selecting a class unfolds related SHACL constraints and violation groups in the NLD and filters the table to the same focus nodes.

The hierarchy omits relations across classes. Users can analyze inter-class context and shape connections in the NLD. Deep or broad trees can still introduce scalability issues, but search and selective expansion help mitigate clutter.

### 5.3 Projection view

The Projection View supports the discovery of clusters of focus nodes sharing the same violations by down-projecting them to 2D. We compute a 2D projection of violating focus nodes during preprocessing using UMAP (McInnes et al., 2018). Our preprocessing script supports two suitable feature sets for this projection: either only violation information (which constraints a focus node violates) or full instance features, including violations. This projection implicitly leads to dense clusters of similar violations (T3). Using full instance features can reveal subclusters within a shared violation pattern when focus nodes differ in fine-grained triggering values (e.g., the same constraint violated via different referenced objects), to identify distinct upstream causes. Conversely, projecting only violation features yields a cleaner separation by violated constraints, whereas instance features may muddy these clusters. More sophisticated projection strategies, e.g., staged coarse-to-fine projections, to first separate by violated constraints and then refine by instance features, could further improve this.

In the projection scatterplot, we show labels of focus node names when space allows. Additional labels appear when zooming in. Users can pan and zoom, and brush rectangular regions to select clusters or subsets.

For the toy dataset, after projecting only the violation information, four salient clusters emerge: Hobbits, regions, locations, and other characters (see Figure 6B). Brushing any one of them unfolds related SHACL constraints and violation groups in the NLD, classes in the Tree view, and filters the table to the same focus nodes.

Distances in the projection depend solely on violation or instance data and are therefore ontology-agnostic. Users can infer such structural context from the NLD. The overplotting due to label density is mitigated by overlap avoidance and revealing more on zoom, with the downside that only a subset of labels is visible when zoomed out.

### 5.4 Tabular view

While NLDs offer unique strengths for topology-related user tasks, Li et al. (2024) report that users mostly treat them as quick sanity checks. One of their participants described this as “make sure nothing weird is going on”, for example, checking that expected connections are present, and the graph is connected. They also report recurring shortcomings once graphs get dense. NLDs do not scale and quickly become “hairballs”, and users can trust the analysis results less when they are presented as a topological visualization. Based on their interviews, Li et al. argue that KG Consumers tend to prefer tables because they are simple, familiar, and domain-agnostic, and because they often make the task at hand straightforward (e.g., data retrieval, ranking, filtering). This efficacy of tables is also explored by Bartram et al. (2021). We therefore complement the NLD in SHACLens with a Tabular View.

The Tabular View allows users to inspect individual focus nodes that cause violations. Focus nodes can also be sorted and grouped (T3). To support the user tasks of finding co-occurrence patterns (T4) and understanding the distributions of properties (T5), the table presents instance properties alongside violation indicators in an interactive LineUp visualization (Gratzl et al., 2013). To construct the table, we filter the instance data for violating focus nodes and join them with their violations from the SHACL report. The result is tabularized: Rows are violating focus node *subjects*, columns are *predicates*, and cells contain *object* values.

**FIGURE 7:**
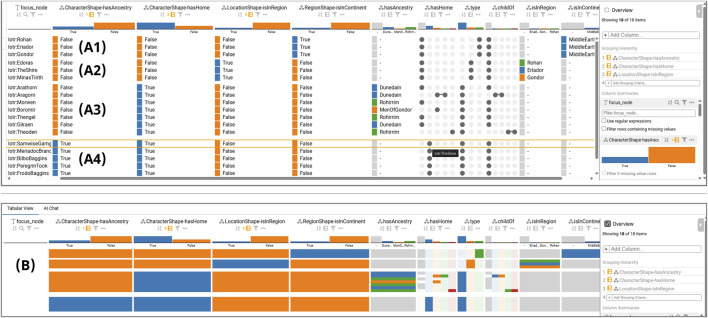
LineUp Tabular view showing a grouping of focus nodes by the constraint violation columns CharacterShape-hasAncestry, CharacterShape-hasHome, LocationShape-isInRegion, and RegionShape- isInContinent. **(A1)** Region group. **(A2)** Location group. **(A3)** Non-Hobbit character group. **(A4)** Hobbit character group. For the Hobbit group, it can be observed that all characters violate both CharacterShape-hasAncestry and CharacterShape-hasHome, none have a value for the hasAncestry column, and all have TheShire for the hasHome column. **(B)** LineUp in overview mode.

The table uses type-aware columns with appropriate inline summaries, including distributions for numerical data, colored bars for categorical data, and UpSet visualizations for set-type data (Lex et al., 2014). Users can sort by multiple columns, filter by ranges or sets, group by columns, search, and show/hide irrelevant columns. LineUp’s overview mode, which condenses a single row down to a height of one pixel, allows users to scan hundreds of rows at once to spot correlation patterns, as shown in Figure 7B. Because of these features, in our evaluation, most large-scale violation analysis was conducted in the Tabular View to spot correlations between instance properties and violation indicators.

Figure 7A shows a grouping by the CharacterShape-hasAncestry constraint, which is violated by the Hobbits group. Even without looking at focus node IDs, which in this case consist of character names, the missing Hobbit ancestry common to all these nodes could be inferred from their common hasHome value “The Shire” with the necessary domain knowledge.

The tabular view suffers from the general difficulty of tabularizing graph data. Depending on the set of focus nodes, many predicates might not apply to all nodes, yielding blank cells and wide tables. For instance, locations naturally lack hasAncestry. We address this issue by allowing users to hide entire columns that are uninteresting for their current analysis. While the table offers many useful features for analyzing focus nodes, the ontology is not directly encoded within the table. As mentioned above, in case these topological tasks become necessary, users can still infer the structural context from the linked NLD.

### 5.5 LLM chat

To support users in navigating and understanding the various views, we developed an integrated LLM Chat. The LLM Chat is connected directly to the application’s internal API, providing access to relevant data and interaction states across the dashboard’s multiple views. This integration enables users to utilize natural language interactions for three primary purposes: (*i*) performing analytical operations equivalent to mouse-driven interactions; (*ii*) obtaining detailed explanations of constraint violations and their underlying causes, and (*iii*) receiving summaries and explanations of the current state and selections within SHACLens.

**FIGURE 8:**
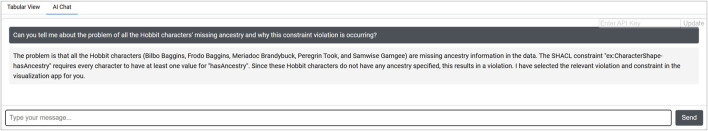
The LLM chat interface. A user asks the LLM to explain the Hobbit characters’ missing ancestry violation. The LLM lists all the relevant focus nodes and explains exactly what the problem is. It uses the app’s API to select those focus nodes, propagating the selection to the other views as if the user had selected them manually.

For instance, users may query the system with questions such as “Can you explain why all Hobbit characters are missing ancestry information and why this violation occurs?”. Internally, the LLM retrieves necessary data via exposed functions, interprets the query contextually, and formulates an appropriate response (see Figure 8). Additionally, the interface synchronizes selections and interactions across all visualizations. In the given example, the LLM automatically selects the affected Hobbit focus nodes, filters the Tabular View accordingly, highlights the corresponding cluster in the projection scatterplot, and expands relevant ontology structures, SHACL constraints, and violation groups within the NLD.

While the depth and accuracy of these explanations depend on the capabilities of the employed LLM and the data complexity and violations in question, the modular design explicitly facilitates the integration and replacement of different LLMs, allowing straightforward updates to newer, more advanced models as they become available through ongoing Machine Learning research.

## 6 Implementation

We implemented SHACLens as a web application combining a React-based frontend and a Python backend. The frontend uses Redux for state management and integrates LineUp for the Tabular View. Based on literature reviews we considered Ontosphere, Protégé, and Cytoscape.js for the NLD, but we ultimately adopted D3.js (Bostock et al., 2011) to gain finer control over the visual representation, force-directed layouts, and custom interaction patterns. The scatterplot projection is also rendered with D3.js, with UMAP applied during preprocessing to embed each violating focus node in two dimensions. On the backend, a FastAPI service ingests the three input KGs–ontology, instance data, and SHACL violation reports. In a pre-processing step, we perform union operations and tabularization. During this stage, we compute the UMAP embeddings and assemble lookup tables that map focus nodes to their classes, constraints, and violation groups, supporting rapid filtering and highlighting in the frontend. The integrated chat interface is built with LangChain and connects directly to frontend APIs, allowing the chosen LLM (GPT-4.1 at the time of writing) to both query the processed KG data and programmatically manipulate selection states as if a user had clicked in the UI. For smaller toy datasets, the model can ingest entire RDF graph files directly; for larger graphs, it relies on summarized metadata served by the backend. This architecture permits straightforward replacement of the LLM. The code for our prototype of SHACLens is available at https://github.com/CursedSeraphim/bikg_app/.

## 7 Case study

We evaluate our approach through a qualitative study based on a transcriptomics knowledge graph from our industry partner containing 5,203 violating focus nodes. The vast majority of focus nodes each have multiple violations associated with them. The user is a data quality analyst who also performs some provider tasks. In the case study, the user identifies the triggering sources and co-occurrence patterns of violations, as well as additional data-quality issues that are not directly related to constraint violations.

For brevity, we omit the first parts of long property shape names and indicate the omission with a diamond. For instance, omics:PrimaryCellSpecimenShape-hasCellType becomes ⋄ hasCellType. Many instances in the KG used for this case study also have hash-like unique IDs. These instances typically have human-readable labels associated with them, which are also stored in the KG. For example, “*Homo sapiens*” is defined as a ⋄ prefLabel property of one node towards the bottom left of the NLD shown in Figure 13. To improve readability, we often refer to the node itself by its human-readable label rather than its actual ID.

### 7.1 Cluster analysis with the projection and Class Tree View

**FIGURE 9:**
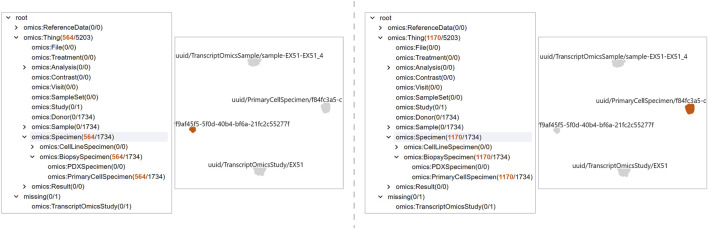
Two distinct clusters emerge for the ⋄ PrimaryCellSpecimen class. The Projection View reveals two subsets of co-occurring violations, shown here by selecting the smaller cluster with 564 focus nodes (left) and the larger cluster with 1,170 focus nodes (right).

The user starts the analysis by brushing the four clusters in the Projection View. This selection highlights the matching classes and shapes in the Class Tree View and NLD and filters the Tabular View. Cross-checking both views shows that clusters tend to align with classes: ⋄ Donor (1,734), two ⋄ PrimaryCellSpecimen clusters (1,170 and 564), and ⋄ TranscriptOmicsSample (1,734). Interestingly, ⋄ PrimaryCellSpecimen does not form a single cluster, but splits into two subsets (see Figure 9). The Class Tree also exposes a single outlier: When the user selects ⋄ Study, the same node is highlighted as belonging to the ⋄ TranscriptOmicsStudy class, which is missing in the ontology. This inspection and brushing supported the user in gaining an overview of the co-occurring violations and relating them to their corresponding class structure. Such an insight would have been difficult to obtain from thousands of entries in an HTML SHACL report. It further serves as a filter and entry point for detail-on-demand in the following exploration.

### 7.2 Detail and cluster comparison in the tabular view

**FIGURE 10:**
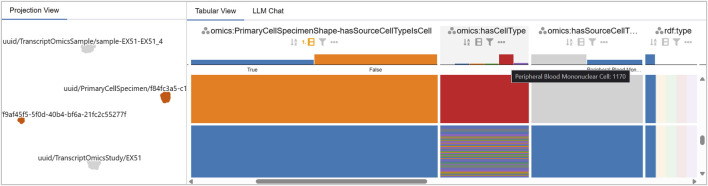
Comparison of the two ⋄ PrimaryCellSpecimen clusters in the LineUp View. In overview mode, grouping by the violated constraint highlights the single difference between clusters: ⋄ hasSourceCellTypeIsCell. The larger cluster (1,170 focus nodes) lacks a ⋄ hasSourceCellType and does not violate the constraint, while the smaller cluster (564 focus nodes) consistently references “Peripheral Blood Mononuclear Cell” as ⋄ hasSourceCellType, triggering the violation.

After performing a selection in the Projection View, the Tabular View is already filtered to the corresponding focus nodes. The user groups rows by *violated constraint* and investigates the referenced object and ⋄ type columns. The two ⋄ PrimaryCellSpecimen clusters differ by a single constraint: cluster A shows four violated constraints, including ⋄ hasCellType, whereas cluster B shows the same four plus ⋄ hasSourceCellTypeIsCell (see Figure 10), an edge that never appears for the focus nodes in cluster A.

Across all sets of co-occurring violations, the referenced objects are almost always identical–e.g., the violating object is always “*Homo sapiens*” for ⋄ hasSpecies and always “normal” for ⋄ hasDisease. Only for ⋄ hasCellType, five different object values appear: Classical Monocyte, Intermediate Monocyte, Non-classical Monocyte, Peripheral Blood Mononuclear Cell, Plasmacytoid Dendritic Cell (see Figure 10).

**FIGURE 11:**
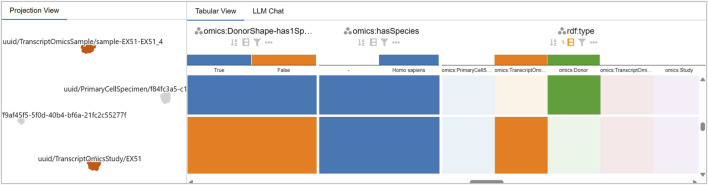
Comparison of ⋄ hasSpecies references across focus nodes. Focus nodes referencing “*Homo sapiens*” only trigger a violation for the ⋄ Donor class, as the corresponding constraint is defined for omics:DonorShape-has1Species but not for ⋄ TranscriptOmicsSample.

Comparing the column listing these objects with the type column, it becomes apparent that they only trigger a violation when used for nodes of class ⋄ Donor. However, when they appear on nodes of class ⋄ TranscriptOmicsSample, no violation is reported. The user investigates the list of constraints in the table and discovers a schema gap where this constraint is not defined for the ⋄ TranscriptOmicsSample class. The user discovers another such schema gap where the constraint ⋄ has1Species is only defined for ⋄ Donor but not ⋄ TranscriptOmicsSample, and therefore, “*Homo sapiens*” nodes only trigger violations when linked to ⋄ Donor nodes (see Figure 11).

**FIGURE 12:**
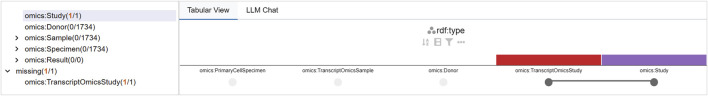
Outlier node typed as both ⋄ Study and ⋄ TranscriptOmicsStudy. The Class Tree exposes ⋄ TranscriptOmicsStudy as missing from the ontology file, LineUp confirms that both class assignments belong to the same focus node, and the UpSet column confirms that the node has two classes.

Finally, the UpSet view also shows the previously spotted outlier node typed as both ⋄ Study and ⋄ TranscriptOmicsStudy (see Figure 12).

Following the analysis with the LineUp view, the user identifies the complete set of object values that trigger violations for each constraint. Without the need for SPARQL queries, the view supported the user in identifying the single differing constraint between clusters. It also helped in summarizing and comparing object values against the ⋄ type column to reveal the patterns driving violations.

### 7.3 Identifying causes in the NLD

**FIGURE 13:**
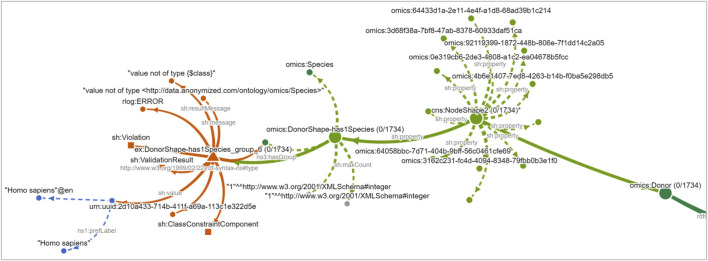
Expansion of a violation path in the NLD for ⋄ has1Species. From right to left, the view shows the ⋄ Donor class, its NodeShape grouping all associated constraints, the violated Property Shape ⋄ has1Species encoding the constraint “must have exactly one object of type omics:Species”, the violation group summarizing 1,734 focus nodes, and the shared violating object. The object “*Homo sapiens*” (blue hexagons and labels on the left) is not connected to any class and is missing the required link to omics:Species (dark green, top center).

**FIGURE 14:**
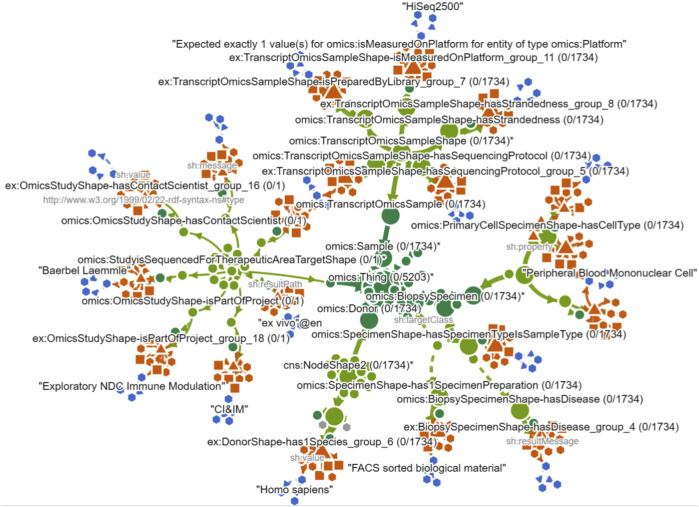
The NLD showing all expanded violation paths in overview. Ontology classes are shown in dark green, SHACL shapes in light green, violation groups in red, and violating instance objects lacking class links in blue.

Moving on to the NLD, the user performs the *expand violation path* action on a class node to reveal nested shapes and violation groups (see Figure 13). This exposes the paths: Class → Node Shape → Property Shapes → Violation Groups. Then, expanding one of the violation groups exposes the instance node identified as a problematic value for this group. This inspection shows that these values lack a typing edge to the required class—e.g., for focus nodes of class ⋄ Donor, one violation group reports ⋄ hasSpecies → “*Homo sapiens*” causes a problem, and the user sees that there is no edge connecting it back to the corresponding class node. I.e., (“*H. sapiens*”, ⋄ type, ⋄ Species), is missing. The same lack of a definition is found for the other violations pertaining to disease, library, strandedness, etc (see Figure 14 for an overview of the full graph). In this manner, the user discovers the causes of all of the violations in this dataset. Again, without the use of SPARQL queries, the NLD supported the user in identifying the exact cause of violations, summarized across violation groups.

### 7.4 LLM assistance and drafting a solution

**FIGURE 15:**
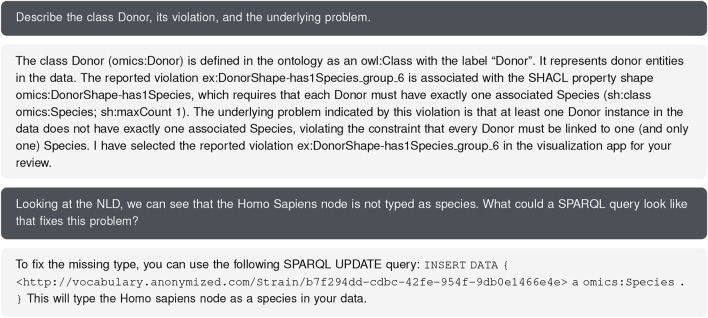
Excerpt of the chat with the LLM, showing problem description and suggested fix. Note that the node itself is not named “*Homo Sapiens*”, but has a link to this label, which the LLM correctly reflected in its update query.

The user is interested in a natural language explanation of the ⋄ Donor problem and sends a prompt requesting a general description of the problem involving ⋄ Donor nodes. The LLM responds with an explanation of the ⋄ has1Species violation, providing RDF snippets for reference, and synchronizes the visual state by selecting ⋄ Donor and its related elements across views. The user then asks for a sketch of a query to fix the problem with the “*Homo Sapiens*” node. The interface returns a possible SPARQL update and a textual explanation. The user then manually reviews and considers the query to address the issue. The exact prompts and responses can be seen in Figure 15. The LLM, therefore, helped the user derive missing information as a step toward supporting the *development of a correction query*, even though we did not target this action of data quality analysts directly.

### 7.5 Summary of findings

Across all clusters, the violations follow the same missing object type pattern, where the object referenced by the violation is never found in the instance data. There are five violation combination patterns as the focus nodes come from four classes, with one class splitting into the two patterns with or without ⋄ hasSourceCellType. Each pattern has a set of constraints violated by recurring underspecified objects. Furthermore, two SHACL definition problems were discovered: There is a schema gap where the constraint ⋄ hasSourceCellType should be defined for ⋄ TranscriptOmicsSample. As a result, the underspecified ⋄ CellType objects do not violate constraints for subjects of this class. A similar schema gap was discovered for the constraint ⋄ has1Species, which is only defined for ⋄ Donor but not for ⋄ TranscriptOmicsSample. Finally, one ontology problem was discovered: The ontology does not define the class ⋄ TranscriptOmicsStudy, which appears in the instance data. To address these issues, it is necessary to add the missing object definitions to the instance data and the missing constraints and class to the ontology.

## 8 Discussion

During the development of SHACLens and its case study, we derived several lessons that can be generalized beyond this work, which we summarize below.

### 8.1 Workflow

#### 8.1.1 Issue- and action-oriented grouping facilitates curation

For both scalability and effectiveness, we found the most crucial part of creating an improved workflow to be equivalence-based grouping, with the aim of surfacing the smallest set of corrective actions or root issues. In the case of SHACLens, this meant grouping thousands of violations by properties that defined the handful of upstream causes that produced them, and directly supporting the actions *picking violations* and *locating their sources*. Previously, this required scanning HTML lists and iteratively writing SPARQL queries. The LLM explanations could sometimes give exact descriptions of the violation sources as well.

#### 8.1.2 LLMs can assist in suggesting corrective actions

Following the previous lesson, the ideal solution would automate the smallest complete set of corrective actions. In cases where this is not possible, or where a human-in-the-loop is desired, not just surfacing root issues, but presenting a suggestion of the precise corrective actions is the next most beneficial step. While SHACLens does not directly address *fixing errors* or allow live data editing, the LLM’s ability to sketch correction queries represents a step towards alleviating the need for analysts to *develop correction queries*. In some cases, the integrated LLM could perfectly explain violations and suggest working correction queries. However, it still suffers from well-known problems, including occasional hallucinations, brittle phrasing, and privacy concerns. This gives rise to the following lesson learned.

#### 8.1.3 Users want to perform sanity checks

Some situations still required double-checking details in the representations and environments that users are most familiar with. In the case of KGs, on the one hand, this means providing an NLD for sanity checks of expected connectedness. On the other hand, users still brought up original RDF files for an occasional fallback for validation. This indicates that users with sufficient expertise still want to see the underlying data and this should be integrated into workflows directly.

#### 8.1.4 Solutions must leave place for serendipitous discovery

The case study demonstrated that the workflow supported both goal-oriented analysis of violations and their triggers, as well as serendipitous open-ended discovery, where the user identified outliers and schema gaps. In general, we see that even highly goal-driven workflows benefit from leaving room for serendipitous findings where users encounter issues or patterns they were not explicitly searching for.

### 8.2 Design

#### 8.2.1 Root-cause evidence should be encoded directly

Despite its scalability drawbacks, the NLD remained necessary to pinpoint the exact cause of topological issues, as it encodes the graph structure most directly. More generally, while different views can help with other analysis goals, such as detecting patterns or groups of issues, root-cause analysis requires a view that directly encodes the underlying evidence, even if it is less effective for other tasks.

#### 8.2.2 Additional interactions can mitigate navigation challenges in NLDs

The act of expanding and collapsing entire subtrees in search of a specific node or relation is an impediment in NLDs. The user reported that hover previews ease this navigation. NLDs may also contain many nodes and relations irrelevant to the user task. To take advantage of this and reduce clutter, the blacklisting feature requested by the user helped focus on the exact root-cause evidence. However, large expanded graphs and long labels can still exceed the screen space, forcing users to keep context in memory, and force-directed layouts remain difficult to control when not frozen. In response to this, a one-click expansion of paths between classes and violations proved highly useful during goal-oriented analysis, as it removed the need to manually navigate cluttered subtrees. More generally, when an interface exposes a large data structure through progressive drill-down interactions, it helps to couple interaction with the user’s task and provide task-driven expansion actions that surface only the most relevant evidence for the current question.

#### 8.2.3 Some representations better support issue-oriented grouping

LineUp was most effective for comparing clusters and spotting recurring patterns, such as the same object repeatedly triggering violations. These correlations also helped uncover schema gaps. The Projection View served as a useful entry point for identifying violation clusters. These views share a strong visual grouping principle via proximity and similarity. For instance, when using LineUp’s overview, grouping by violations, and spotting which instance feature values end up in the same groups.

#### 8.2.4 Unfamiliar representations can challenge users

However, tabularized graph data was not always intuitive for users accustomed to graph-based reasoning. The row–column–cell reading of subject, predicate, and object can be unintuitive when the underlying mental model is triple- or edge-centric. This finding poses interesting questions for future work regarding the effectiveness of tables for data workers with different levels of expertise Bartram et al. (2021). More broadly, when a design re-encodes information in an unfamiliar way to better support a task, this diversion from established conventions can cause confusion.

#### 8.2.5 Visualizations can invite broader user groups to engage with complex data

One additional takeaway from the user was their expressed interest in using visualizations across many more subtasks in their work processes, as well as their view that visualizations are highly effective for representing data and for bridging the gap to non-experts, such as the data providers.

#### 8.2.6 Scalability and generalizabilty remain challenging in KG visualization

SHACLens was succesfully applied to a dataset containing 5,203 violating nodes, but some scalability issues remain: LineUp’s overview mode is constrained by screen height, the NLD remains overwhelming when expanding many subtrees, and the tabularization and embedding preprocessing steps are slow for real-time use. While our solution generalizes to KGs of different domains, it is most effective when violations cluster around recurring patterns. Highly idiosyncratic error patterns could pose generalizability challenges, as the richness of OWL ontologies and SHACL constraints makes it hard to anticipate them.

### 8.3 Future work

While the case study demonstrates that SHACLens can surface violation patterns, there are various potential avenues for further enhancements.

Explanations could be made verifiable by linking view contents and selections to concrete triples and lines in the original RDF or.ttl files through a synchronized source panel. This would combine the visualization with evidence and streamline the occasional validation process. In privacy-constrained settings, local, potentially fine-tuned models tailored to SHACL and the ontology in use could be considered. Graph-based Retrieval-Augmented Generation (RAG) (Lewis et al., 2020) could be applied, sending queries to the RDF data back and forth, such that even for large graphs, the LLM could fit the relevant RDF within its context window.

The Projection View could show multiple comparative embeddings derived from different feature sets. Cluster summary and difference visualizations via small cluster insets that list dominant violated shapes, common object values, and classes could provide instant context of the clusters even before any user selection. Especially for large datasets, progressively refined summary visualizations at coarse zoom that resolve to points on demand could further improve this.

The NLD could also benefit from semantic zoom combined with the application of concepts like aggregation + simplification, overview + detail, and focus + context. For instance, it could display super-nodes and violation group summaries during coarse overview, and smoothly expand down to individual violations and even instance data. Context preservation could be improved via a minimap or more sophisticated strategies, such as out-of-view visualizations by pinning critical subgraphs.

In case of non-human-readable node IDs, more intelligible identifiers could be automatically inferred from relevant triples. For instance, for cryptic constraint labels, the node shape target class together with the exact constraints could be transformed into a label.

The workflow could be further operationalized by a “what-if” mode that virtually applies hypothetical fixes and previews their effects, allowing a user to tick them off one by one. Alternatively, it could directly operate on live data together with assisting the *develop correction query* and *verify correction* user actions.

## 9 Conclusion

Validation of knowledge graphs through SHACL constraints can produce violation reports that, at industry scale, are too large to inspect manually. In collaboration with pharmaceutical experts, we analyzed their existing violation analysis workflow, identified user actions and tasks, and targeted the two early user actions: *picking violations* and *locating their source*. We introduced SHACLens, a visualization workflow that applies multiple coordinated views to analyze SHACL violation reports in knowledge graphs. It combines a violation-aware NLD, a Class Tree, a projection of violating focus nodes, a LineUp table of their detailed instance data, and an LLM interface. The workflow is ontology-agnostic and is most effective when violations cluster into recurring patterns. In a transcriptomics case study with 5,203 violating focus nodes, the workflow supported a goal-oriented analysis loop: Selecting clusters of co-occurring violations, inspecting correlation patterns, and tracing detailed causes. It revealed triggering sources such as missing object types and, through serendipitous open-ended exploration, surfaced ontology and SHACL definition issues. We discussed design lessons, including the value of violation grouping, quick expansion of deep analysis-relevant paths in the NLD to mitigate navigation overhead, and the limits of each component. The user interaction with SHACLens further suggested that, for occasional validation and cross-checking, access to a source view of the original data could be a particularly valuable feature for domain experts.

## Data Availability

The datasets presented in this article are not readily available because the transcriptomics dataset used in the case study is proprietary. However, the dataset used as a guiding example throughout the paper to illustrate the methods is available in the public repository and in the deployed application, both of which are linked in the manuscript. Requests to access the datasets should be directed to christian.steinparz@jku.at.

## References

[B1] Alani H. (2003). “TGVizTab: an ontology visualisation extension for protégé,” in Knowledge capture, workshop on visualization information in knowledge engineering, 6.

[B2] Alom H. (2022). A library for visualizing SHACL over knowledge graphs. Master’s Thesis, Gottfried Wilhelm Leibniz Univ. Hann. Hann. Ger. 10.15488/11944

[B3] Bartram L. Correll M. Tory M. (2021). Untidy data: the unreasonable effectiveness of tables. IEEE Trans. Vis. Comput. Graph. 28, 686–696. 10.1109/TVCG.2021.3114830 34591767

[B4] Bikakis N. Sellis T. (2016). Exploration and visualization in the web of big linked data: a survey of the state of the art. ArXiv:1601.08059, 1558. 10.48550/arXiv.1601.08059

[B5] Bosca A. Bonino D. Pellegrino P. (2005). “OntoSphere: more than a 3D ontology visualization tool,” in Swap, 15.

[B6] Bostock M. Ogievetsky V. Heer J. (2011). D^3^ data-driven documents. IEEE Transactions Visualization Computer Graphics 17, 2301–2309. 10.1109/TVCG.2011.185 22034350

[B7] Brickley D. Guha R. (2014). *RDF schema 1.1*. W3C recommendation. World Wide Web Consort. Available online at: https://www.w3.org/TR/rdf-schema/.

[B8] Crisan A. Fiore-Gartland B. Tory M. (2021). Passing the data baton: a retrospective analysis on data science work and workers. IEEE Trans. Vis. Comput. Graph. 27, 1860–1870. 10.1109/TVCG.2020.3030340 33048684

[B9] Dudáš M. Zamazal O. Svátek V. (2014). “Roadmapping and navigating in the ontology visualization landscape,” in Knowledge engineering and knowledge management. Editors Janowicz K. Schlobach S. Lambrix P. Hyvönen E. (Cham: Springer International Publishing), 137–152. 10.1007/978-3-319-13704-9_11

[B10] Dudáš M. Lohmann S. Svátek V. Pavlov D. (2018). Ontology visualization methods and tools: a survey of the state of the art. Knowl. Eng. Rev. 33, e10. 10.1017/S0269888918000073

[B11] Ehrlinger L. Wöß W. (2016). Towards a definition of knowledge graphs. In Joint proceedings of the posters and demos track of the 12th international conference on semantic systems (SEMANTiCS 2016) and the 1st international workshop on semantic change and evolving semantics (SuCCESS ’16), eds. Martin M. Cuquet M. Folmer E. 1695 Leipzig, Germany: CEUR-WS.org. Available online at: https://ceur-ws.org/Vol-1695/.

[B12] Falconer S. (2010). Ontograf. Available online at: https://protegewiki.stanford.edu/wiki/OntoGraf (Accessed August 04, 2024).

[B13] Figuera M. Rohde P. D. Vidal M.-E. (2021). “Trav-SHACL: efficiently validating networks of SHACL constraints,” in Proceedings of the Web Conference 2021, 3337–3348.

[B14] Ghadiri Bashardoost B. Christodoulakis C. Hassas Yeganeh S. Miller R. J. Lyons K. Hassanzadeh O. (2015). “VizCurator: a visual tool for curating open data,” in Proceedings of the 24th International Conference on World Wide Web, 195–198.

[B15] Gratzl S. Lex A. Gehlenborg N. Pfister H. Streit M. (2013). LineUp: visual analysis of multi-attribute rankings. IEEE Trans. Vis. Comput. Graph. (InfoVis ’13) 19, 2277–2286. 10.1109/TVCG.2013.173 24051794 PMC4198697

[B16] Horridge M. (2010). OWLViz. Available online at: https://protegewiki.stanford.edu/wiki/OWLViz (Accessed August 04, 2024).

[B17] Hoyt C. T. Domingo-Fernández D. Aldisi R. Xu L. Kolpeja K. Spalek S. (2019). Re-curation and rational enrichment of knowledge graphs in biological expression language. Database 2019, baz068. 10.1093/database/baz068 31225582 PMC6587072

[B18] Huaman E. Fensel D. (2022). “Knowledge graph curation: a practical framework,” Proceedings of the 10th International Joint Conference on Knowledge Graphs New York, NY, USA: Association for Computing Machinery, 166–171. 10.1145/3502223.3502247

[B19] Katifori A. Halatsis C. Lepouras G. Vassilakis C. Giannopoulou E. (2007). Ontology visualization methods—a survey. ACM Comput. Surv. 39, 10–es. 10.1145/1287620.1287621

[B20] Klímek J. Škoda P. Nečaský M. (2019). Survey of tools for linked data consumption. Semantic Web 10, 665–720. 10.3233/SW-180316

[B21] Knublauch H. Kontokostas D. (2017). *Shapes constraint language (SHACL)*. W3c recommendation. World Wide Web Consort. (W3C). Available online at: https://www.w3.org/TR/shacl/.

[B22] Koutrika G. Lakshmanan L. V. Riedewald M. Stefanidis K. (2014). “Exploratory search in databases and the web,” in EDBT/ICDT workshops, 158–159.

[B23] Kunowski P. Boiński T. (2012). SOVA (simple ontology visualization API). Available online at: https://protegewiki.stanford.edu/wiki/SOVA (Accessed August 04, 2024).

[B24] Lewis P. Perez E. Piktus A. Petroni F. Karpukhin V. Goyal N. (2020). Retrieval-augmented generation for knowledge-intensive NLP tasks. Adv. Neural Information Processing Systems 33, 9459–9474. 10.5555/3495724.3496517

[B25] Lex A. Gehlenborg N. Strobelt H. Vuillemot R. Pfister H. (2014). UpSet: visualization of intersecting sets. IEEE Transactions Visualization Computer Graphics 20, 1983–1992. 10.1109/TVCG.2014.2346248 26356912 PMC4720993

[B26] Li H. Appleby G. Brumar C. D. Chang R. Suh A. (2024). Knowledge graphs in practice: characterizing their users, challenges, and visualization opportunities. IEEE Trans. Vis. Comput. Graph. 30, 584–594. 10.1109/TVCG.2023.3326904 38096099

[B27] Liepinš R. Grasmanis M. Bojars U. (2014). “OWLGrEd ontology visualizer,” in Proceedings of the 2014 international conference on developers (Riva del Garda, Italy: CEUR-WS.org), 37–42. Available online at: https://ceur-ws.org/Vol-1268/.

[B28] Mäkelburg J. Zacouris Z. Ke J. Acosta M. (2025). “SHACL dashboard: analyzing data quality reports over large-scale knowledge graphs,” in International Semantic Web Conference (Springer), 96–112.

[B29] Malik K. M. Krishnamurthy M. Alobaidi M. Hussain M. Alam F. Malik G. (2020). Automated domain-specific healthcare knowledge graph curation framework: Subarachnoid hemorrhage as phenotype. Expert Syst. Appl. 145, 113120. 10.1016/j.eswa.2019.113120

[B30] McCusker J. P. Rashid S. M. Agu N. Bennett K. P. McGuinness D. L. (2018). “The whyis knowledge graph framework in action,” in ISWC (P&D/Industry/BlueSky), 4.

[B31] McInnes L. Healy J. Melville J. (2018). UMAP: uniform manifold approximation and projection for dimension reduction. J. Open Source Softw. 3 (29), 861. 10.21105/joss.00861

[B32] Nobre C. Meyer M. Streit M. Lex A. (2019). The state of the art in visualizing multivariate networks. Comput. Graph. Forum 38, 807–832. 10.1111/cgf.13728

[B33] Noy N. Gao Y. Jain A. Narayanan A. Patterson A. Taylor J. (2019). Industry-scale knowledge graphs: lessons and challenges: five diverse technology companies show how it’s done. Queue 17, 20–48. 10.1145/3329781.3332266

[B34] OpenAI Achiam J. Adler S. Agarwal S. Ahmad L. Akkaya I. (2024). GPT-4 technical report.

[B35] Paciello R. Trani L. Bailo D. Vinciarelli V. Sbarra M. (2023). “Shapeness: a shacl-driven metadata editor,” in Metadata and semantic research. Editors Garoufallou E. Vlachidis A. (Cham: Springer Nature Switzerland), 274–288.

[B36] Po L. Bikakis N. Desimoni F. Papastefanatos G. (2020). Linked data visualization: techniques, tools, and big data, 10. Springer.

[B37] Radford A. Narasimhan K. (2018). Improving language understanding by generative pre-training. Tech. Rep. Open AI. Available online at: https://cdn.openai.com/research-covers/language-unsupervised/language_understanding_paper.pdf.

[B38] Simsek U. Angele K. Kärle E. Opdenplatz J. Sommer D. Umbrich J. (2021). “Knowledge graph lifecycle: building and maintaining knowledge graphs,” in Second international workshop on knowledge graph construction, 16.

[B39] Sintek M. (2007). OntoViz. Available online at: https://protegewiki.stanford.edu/wiki/OntoViz (Accessed August 04, 2024).

[B40] Spahiu B. Maurino A. Palmonari M. (2018). “Towards improving the quality of knowledge graphs with data-driven ontology patterns and SHACL,” in ISWC (best workshop papers), 103–117.

[B41] Storey M.-A. Musen M. Silva J. Best C. Ernst N. Fergerson R. (2001). “Jambalaya: interactive visualization to enhance ontology authoring and knowledge acquisition in protégé,” in Workshop on interactive tools for knowledge capture (citeseer), 73.

[B42] TopQuadrant, Inc (2019). TopBraid composer. Available online at: https://topbraidcomposer.org/html/(Accessed August 04, 2024).

[B43] Víctor Méndez R. M. (2024). NeOn-Toolkit plugin for OWL ontology visualization. Available online at: http://neon-toolkit.org/wiki/1.x/OWL_Ontology_Visualization.html (Accessed August 04, 2024).

[B44] Vrandečić D. Krötzsch M. (2014). Wikidata: a free collaborative knowledgebase. Commun. ACM 57, 78–85. 10.1145/2629489

[B45] Wilkinson M. D. Dumontier M. Aalbersberg I. J. Appleton G. Axton M. Baak A. (2016). The FAIR guiding principles for scientific data management and stewardship. Sci. Data 3, 160018. 10.1038/sdata.2016.18 26978244 PMC4792175

[B46] World Wide Web Consortium (2014). *RDF 1.1 concepts and abstract syntax*. W3C recommendation REC-rdf11-concepts-20140225. World Wide Web Consort. Available online at: https://www.w3.org/TR/2014/REC-rdf11-concepts-20140225/.

[B47] Yahya M. Berberich K. Ramanath M. Weikum G. (2016). Exploratory querying of extended knowledge graphs. Proc. VLDB Endow. 9, 1521–1524. 10.14778/3007263.3007299

